# Performance of Large Language Models on Medical Oncology Examination Questions

**DOI:** 10.1001/jamanetworkopen.2024.17641

**Published:** 2024-06-18

**Authors:** Jack B. Longwell, Ian Hirsch, Fernando Binder, Galileo Arturo Gonzalez Conchas, Daniel Mau, Raymond Jang, Rahul G. Krishnan, Robert C. Grant

**Affiliations:** 1 Princess Margaret Cancer Centre, University Health Network, Toronto, Ontario, Canada; 2 Department of Medicine, University of Toronto, Toronto, Ontario, Canada; 3 Institute of Medical Science, University of Toronto, Toronto, Ontario, Canada; 4 Department of Computer Science, University of Toronto, Toronto, Ontario, Canada; 5 Department of Laboratory Medicine and Pathobiology, University of Toronto, Toronto, Ontario, Canada; 6 Vector Institute, Toronto, Ontario, Canada; 7 ICES, Toronto, Ontario, Canada

## Abstract

**Importance:**

Large language models (LLMs) recently developed an unprecedented ability to answer questions. Studies of LLMs from other fields may not generalize to medical oncology, a high-stakes clinical setting requiring rapid integration of new information.

**Objective:**

To evaluate the accuracy and safety of LLM answers on medical oncology examination questions.

**Design, Setting, and Participants:**

This cross-sectional study was conducted between May 28 and October 11, 2023. The American Society of Clinical Oncology (ASCO) Oncology Self-Assessment Series on ASCO Connection, the European Society of Medical Oncology (ESMO) Examination Trial questions, and an original set of board-style medical oncology multiple-choice questions were presented to 8 LLMs.

**Main Outcomes and Measures:**

The primary outcome was the percentage of correct answers. Medical oncologists evaluated the explanations provided by the best LLM for accuracy, classified the types of errors, and estimated the likelihood and extent of potential clinical harm.

**Results:**

Proprietary LLM 2 correctly answered 125 of 147 questions (85.0%; 95% CI, 78.2%-90.4%; *P* < .001 vs random answering). Proprietary LLM 2 outperformed an earlier version, proprietary LLM 1, which correctly answered 89 of 147 questions (60.5%; 95% CI, 52.2%-68.5%; *P* < .001), and the best open-source LLM, Mixtral-8x7B-v0.1, which correctly answered 87 of 147 questions (59.2%; 95% CI, 50.0%-66.4%; *P* < .001). The explanations provided by proprietary LLM 2 contained no or minor errors for 138 of 147 questions (93.9%; 95% CI, 88.7%-97.2%). Incorrect responses were most commonly associated with errors in information retrieval, particularly with recent publications, followed by erroneous reasoning and reading comprehension. If acted upon in clinical practice, 18 of 22 incorrect answers (81.8%; 95% CI, 59.7%-94.8%) would have a medium or high likelihood of moderate to severe harm.

**Conclusions and Relevance:**

In this cross-sectional study of the performance of LLMs on medical oncology examination questions, the best LLM answered questions with remarkable performance, although errors raised safety concerns. These results demonstrated an opportunity to develop and evaluate LLMs to improve health care clinician experiences and patient care, considering the potential impact on capabilities and safety.

## Key Points

**Question:**

What medical oncology knowledge is encoded by large language models (LLMs)?

**Findings:**

In this cross-sectional study evaluating 8 LLMs, proprietary LLM 2 correctly answered 85.0% of examination-style multiple-choice questions from the American Society of Oncology, the European Society of Medical Oncology, and an original set from the authors, outperforming proprietary LLM 1 and open-source models. However, 81.8% of incorrect answers were rated as having a medium or high likelihood of moderate to severe harm if acted upon in practice.

**Meaning:**

These findings suggest that LLMs can accurately answer questions requiring advanced knowledge of medical oncology, although errors may cause harm.

## Teaser

This cross-sectional study evaluates the accuracy of large language model (LLM) answers to examination-style multiple choice medical oncology questions and assessed whether errors in LLM responses would be likely to cause harm.

## Introduction

Large language models (LLMs) have the potential to transform health care.^1,2^ LLMs are deep learning systems trained using massive corpora of text from the internet to predict the next word in a sentence, which are then fine-tuned to perform specific tasks such as answering questions with human-like responses.^3^ Potential applications of LLMs in oncology are broad, ranging from assisting clinicians with administrative tasks or decision-making to interacting with patients to provide medical or emotional counselling.^4,5^

LLMs encode a remarkable amount of medical knowledge without specific training. Just like with medical trainees, we can evaluate the medical knowledge of LLMs using standardized tests. LLMs can pass the US Medical Licensing Examination^6,7^ and provide a rationale for answers that display comprehension of the question, recall of the relevant knowledge, and reasoning to arrive at the solution. However, the performance on examinations across different medical subspecialties has varied.^8,9,10,11,12^

Dynamic and specialized knowledge characterize medical oncology. For example, the average rate of US Food and Drug Administration approvals surged from 7.4 per year from 2000 to 2004 to 56 per year from November 2017 to October 2022.^13^ Knowledge evolves swiftly, and a single trial can change practice compared with all preceding literature. Furthermore, the volume of published knowledge on cancer is vast, with over 3 million PubMed studies about cancer.^14^ Whether the performance of LLMs transfers to medical oncology remains largely unknown, with current research generally limited to specific cancer types^15,16,17,18^ or general questions.^19^

In this study, we evaluated the medical oncology knowledge encoded in LLMs using standardized examination-style questions from the American Society of Clinical Oncology (ASCO), the European Society for Medical Oncology (ESMO), and original examination-style questions created by our team. Our goal was to understand the accuracy and limitations of LLMs applied to medical oncology to guide future research and applications.

## Methods

We conducted this cross-sectional study between May 28 and October 11, 2023. The study was exempt from research ethics board approval and the need for informed consent in accordance with 45 CFR §46, given the lack of involvement of human participants. We followed the Strengthening the Reporting of Observational Studies in Epidemiology (STROBE) reporting guideline for cross-sectional studies.

### Questions

#### ASCO

ASCO’s Oncology Self-Assessment Series on ASCO Connection is a publicly accessible question bank hosted on the organization’s official website.^20^ Questions are multiple-choice with one correct answer and cover various topics. The website provides the correct answer and an explanation referencing pertinent published papers for each question. The bank includes 52 questions, with 49 questions containing 4 multiple-choice options and 4 having 5 options.

#### ESMO

ESMO provides multiple-choice questions with a single correct answer from the previous 2 years of ESMO’s Examination Trial Questions from the ESMO Academy (2021 and 2022, type A).^21^ These questions are “specifically designed for medical oncologists in training who intend to take the ESMO examination. [They are] also suitable for oncology professionals who are in need of a refresher course”^22^. We discarded questions based on images because of the LLM’s capability at the time, leaving 75 of 80 questions for the final set. Of the chosen questions, 74 contained 5 multiple-choice options, and 1 included 4. ESMO provided the right answers, but explanations and references were developed by 2 medical oncologists (I.H. and F.B.).

#### Original Questions

The training data sources used by proprietary LLMs are not publicly available and could theoretically include these examinations. To remove any potential impact of questions from ASCO or ESMO included during the training of the LLMs, we tested performance on unseen original questions. Three oncologists (I.H., F.B., and G.A.G.C.) developed 20 original questions. The questions resembled ASCO and ESMO, maintaining the multiple-choice format, with 16 questions containing 4 options and 4 with 5 options. We justified each answer with references to the literature. One question was miswritten and sent through a more recent LLM.

### Large Language Models

ChatGPT-3.5 (proprietary LLM 1; May 28, 2023 version; OpenAI) and ChatGPT-4 (proprietary LLM 2; May 28, 2023 version; OpenAI) were accessed through ChatGPT’s website.^23^ We presented all questions to proprietary LLM 1 and proprietary LLM 2 from May 28 to July 19, 2023, except the miswritten question, which was presented on October 11, 2023. We labeled the options for the ESMO multiple-choice answers A through E for consistency with ASCO and original questions. LLMs were prompted by copying and pasting the questions and potential answers into the user interface. Additional prompting techniques are described in the eMethods in Supplement 1.

For comparison, we evaluated 6 open-source LLMs with publicly available weights ranked highly on Chatbot Arena^24^: Mistral-7B-Instruct-v0.2,^25^ Mixtral-8x7B-v0.1,^26^ Llama-2-13b-chat,^27^ Nous-Hermes-Llama2-70b,^28^ openchat-3.5-1210,^29^ and BioMistral-7B DARE.^30^ BioMistral-7B DARE is tailored for biomedical domains. Technical details on the use of these LLMs are described in the eMethods in Supplement 1.

### Data Coding

Each response was recorded, including the chosen letter answer and prose explanation. Two oncologists (I.H. and F.B.) classified the prose that accompanied answers into a 4-level ordinal error scale: wrong with major errors, wrong with minor errors, right with minor errors, and right with no errors. Major errors were defined as explanations showing a limited understanding of the core concepts required to answer the question in relation to the scientific and clinical consensus. Minor errors were defined as explanations consistent with an understanding of the core concepts, although the final answer was incorrect. Discussion with a third medical oncologist (R.G.) solved disagreements. Following a previously published framework, the oncologists evaluated answers for agreement with the scientific and clinical consensus, the likelihood and extent of possible harm, reading comprehension, recall of relevant clinical knowledge, and manipulation of knowledge via valid reasoning.^7^ A medical oncologist (R.G.) categorized questions based on the year of the most recent publication needed to arrive at the correct answer. If the question did not require information from a publication after 2018, no specific year was assigned, and these questions were deemed as not requiring recent information. Finally, the oncologists marked questions where the answer provided by ESMO or ASCO may be incorrect in light of more current literature or may reflect regional variations.

### Statistical Analysis

Our primary analysis tested whether the best model provided more accurate answers than random chance alone using the binomial distribution. McNemar test compared the distribution of correct answers between LLMs, while Fisher test compared the distribution of errors within explanations associated with incorrect answers. Weighted κ with linear weights^31^ assessed if there was an agreement between the 2 oncologists evaluating the accuracy of the best model’s explanations. A Wilcoxon rank sum test compared the distribution of reference years among right and wrong answers.^32^ A 2-sided *P* value of .05 indicated statistical significance. We conducted statistical analysis in R version 4.3.0 (R Project for Statistical Computing).^33^

## Results

We evaluated LLMs across 147 examination questions, including 52 from ASCO, 75 from ESMO, and 20 original questions (Table; eTable 1 in Supplement 1). The most common category was hematology (22 of 147 [15.0%]), although the questions covered a broad range of topics. ESMO included more general questions, such as considering the mechanisms and toxic effects of systemic therapies. From the entire question set, 41 of 147 (27.9%) required knowledge of evidence published from 2018 onwards. LLMs provided answers in prose to all questions (Box), with proprietary LLM 2 requiring a prompt to select a specific answer in 33 of 147 questions (22.4%).

**Table.** Characteristics of the Examination-Style Oncology Questions Used to Evaluate Models

Characteristic	No. of questions (%)			
	ASCO Oncology Self-Assessment (n = 52)	ESMO Examination Trial questions (n = 75)	Original questions (n = 20)	Total (N = 147)
Topic				
Breast	6 (4.1)	9 (6.1)	0	15 (10.0)
General/rarer tumor	0	13 (8.8)	1 (0.7)	14 (9.5)
Gastrointestinal	5 (3.4)	10 (6.8)	4 (2.7)	19 (12.9)
Genitourinary	5 (3.4)	9 (6.1)	3 (2.0)	17 (11.6)
Hematology	10 (6.8)	8 (5.4)	4 (2.7)	22 (15.0)
Other	26 (17.7)	26 (17.7)	8 (5.4)	60 (40.8)
Year the most recent evidence required for the correct answer was published				
Before 2018	32 (21.8)	65 (44.2)	9 (6.1)	106 (72.1)
2018	5 (3.4)	6 (4.1)	0	11 (7.5)
2019	5 (3.4)	2 (1.3)	2 (1.3)	9 (6.1)
2020	7 (4.8)	2 (1.3)	6 (4.1)	15 (10)
2021	3 (2.0)	0	1 (0.7)	4 (2.7)
2022	0	0	2 (1.3)	2 (1.3)

Abbreviations: ASCO, American Society of Clinical Oncology; ESMO, European Society for Medical Oncology.

Box. **Two Selected Questions From the ASCO Oncology Self-Assessment, With Response From Large Language Model (LLM) 2**

**Question 1**

A 62-y-old woman is receiving palliative chemotherapy for metastatic breast cancer to the lung and bone. She presents to the clinic before her next round of chemotherapy with sudden-onset shortness of breath and a cough. No one in her home has had similar symptoms. She has not been febrile, and her cough is nonproductive. Two days ago, she traveled by car from her relative’s house, which is six hours away. This morning, she noticed that she had more swelling in her legs than usual and she reports right mid-back pain when she takes a deep breath. A complete blood count shows her white blood count and differential to be within normal limits. A CT angiogram shows right lower lobe pulmonary embolus. Which of the following treatments would be most appropriate?

A. Subcutaneous heparin

B. Warfarin

C. Low molecular weight heparin, followed by oral warfarin

D. Low molecular weight heparin or a direct oral anticoagulant

**Proprietary LLM 2 Response**

The patient’s presentation of sudden-onset shortness of breath, right mid-back pain with deep breath, and leg swelling, along with the finding of a right lower lobe pulmonary embolus on CT angiogram, is suggestive of acute pulmonary embolism. The most likely cause in this patient is venous thromboembolism secondary to her metastatic cancer and recent prolonged travel.

In the setting of cancer, low molecular weight heparin (LMWH) or a direct oral anticoagulant (DOAC) is recommended as first-line treatment for acute venous thromboembolism, including pulmonary embolism. This is due to the fact that cancer patients have a higher rate of recurrent venous thromboembolism and a higher risk of bleeding on warfarin compared to patients without cancer. Therefore, the correct answer is D. Low molecular weight heparin or a direct oral anticoagulant. Subcutaneous heparin and warfarin are not the preferred treatments in this setting. Bridging with LMWH followed by warfarin (option C) is also less preferable because warfarin has a higher risk of bleeding in cancer patients and is less effective in preventing recurrent VTE compared with LMWH or DOAC.

**Question 2**

A 61-y-old woman presents to the oncology clinic for follow-up after completing eight cycles of treatment for right-sided colon cancer metastatic to the liver and lung. She was treated with 5-fluorouracil, leucovorin, and oxaliplatin (FOLFOX) and bevacizumab for 4 mos. Prior CT scans showed a partial response to treatment with reduction in disease burden in her liver and lungs. She reports developing fatigue that prevents her from hiking, as well as tingling neuropathy that prevents her from gardening and preparing meals. Molecular testing of her tumor shows microsatellite stable disease, wild-type RAS, and a *BRAF* V600E mutation.

Which of the following is the most appropriate next step?

A. Continue FOLFOX and bevacizumab

B. Switch to capecitabine and bevacizumab

C. Switch to encorafenib and cetuximab

D. Switch to fluorouracil and irinotecan (FOLFIRI), plus bevacizumab

**Proprietary LLM 2 Response**

The patient in this scenario is experiencing neuropathy, a common side effect of oxaliplatin, a component of the FOLFOX regimen. Oxaliplatin-induced peripheral neuropathy is typically dose-dependent and cumulative, often leading to symptoms such as numbness, tingling, and discomfort in the hands and feet. These side effects can significantly affect the patient’s quality of life and can be an indication to change chemotherapy regimens.

Capecitabine could be an alternative, as it is another form of chemotherapy that does not have neuropathy as a common side effect. However, the presence of the *BRAF* V600E mutation is important to note, as it implies a less favorable prognosis but also opens the opportunity for targeted therapy.

The *BRAF* V600E mutation is found in approximately 10% of colorectal cancers and is associated with a poorer prognosis. Fortunately, targeted therapies are available. A combination of encorafenib (a *BRAF* inhibitor) and cetuximab (an *EGFR* inhibitor) has been shown in clinical trials to improve survival in patients with metastatic colorectal cancer harboring the BRAF V600E mutation.

Therefore, given the patient’s adverse effects with the FOLFOX regimen and the presence of the *BRAF* V600E mutation, the most appropriate next step would be to switch to encorafenib and cetuximab (Choice C).

Abbreviations: ASCO, American Society of Clinical Oncology; CT, computed tomography; VTE, venous thromboembolism.

**Figure 1.**
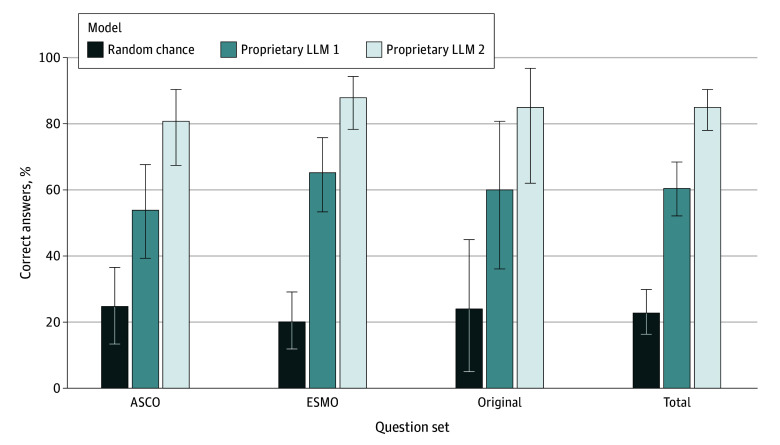
Percentage of Correct Answers Expected by Random Chance and Achieved by 2 Proprietary Large Language Models (LLMs) Error bars indicate 95% CIs. ASCO indicates American Society of Clinical Oncology; ESMO, European Society for Medical Oncology.

Proprietary LLM 2 was most accurate among LLMs, correctly answering 125 of 147 questions (85.0%; 95% CI, 78.2%-90.4%; *P* < .001 vs random answering). Prompt engineering did not enable proprietary LLM 2 to correctly answer any of the questions that were incorrectly answered without specific prompting. Twelve of 22 incorrect answers (54.5%; 95% CI, 32.2%-75.6%) were corrected when proprietary LLM 2 was offered a second attempt. Performance on each of the specific sets of questions was similar, with 42 of 52 on ASCO (80.8%; 95% CI, 67.5%-90.4%; *P* < .001 vs random answering), 66 of 75 on ESMO (88.0%; 95% CI, 78.4%-94.4%; *P* < .001), and 17 of 20 on the original questions (85.0%; 95% CI, 62.1%-96.8%; *P* < .001) (Figure 1).

Proprietary LLM 1 correctly answered 89 of 147 questions (60.5%; 95% CI, 39.5%-67.8%; *P* < .001 vs random answering) of the questions, which was inferior to proprietary LLM 2 (χ^2^_1_ = 24.596; *P* < .001). Mixtral-8x7B-v0.1 was best among open-source LLMs (eFigure in Supplement 1), correctly answering 87 of 147 questions (59.2%; 95% CI, 50.0%-66.4%), which was not significantly different from proprietary LLM 1 (χ^2^_1_ = 0.021; *P* > .88) but was inferior to proprietary LLM 2 (χ^2^_1_ = 23.564; *P* < .001). BioMistral-7B DARE, an LLM tuned for the biomedical domain, answered 50 of 147 questions (33.6%; 95% CI, 26.0%-41.7%) correctly, inferior to both proprietary LLM 1 (χ^2^_1_ = 22.737; *P* < .001) and proprietary LLM 2 (χ^2^_1_ = 60.012; *P* < .001).

**Figure 2.**
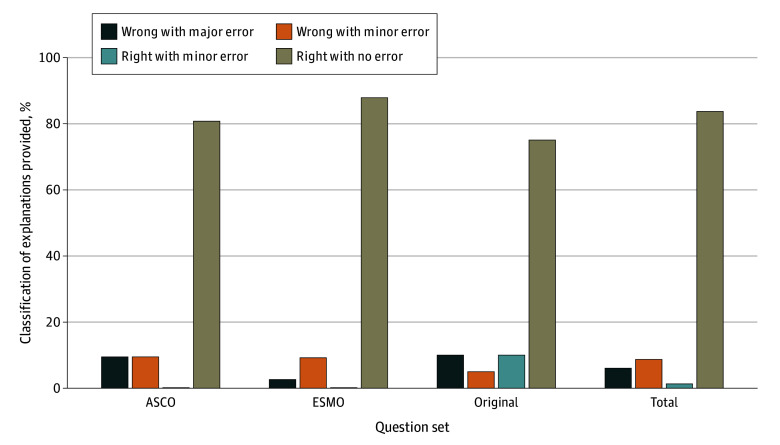
Classifications of Explanations for Answers Provided by Proprietary Large Language Model 2 in Relation to the Scientific and Clinical Consensus ASCO indicates American Society of Clinical Oncology; ESMO, European Society for Medical Oncology.

The prose provided by proprietary LLM 2 answered most questions correctly without errors when evaluated qualitatively by clinicians according to the scientific and clinical consensus (123 of 147 [83.7%]; 95% CI, 76.7%-89.3%) (Figure 2). There was a high concordance between clinician ratings (weighted κ 83.4%; 95% CI, 80.0%-87.0%). Among incorrect answers, 13 of 22 (59.1%; 95% CI, 36.4%-79.3%) contained minor errors, and 9 of 22 (40.9%; 95% CI, 20.7%-63.6%) had major errors. We did not observe any hallucinations, which is when an LLM writes content that cannot be verified or conflicts with facts.^34^

**Figure 3.**
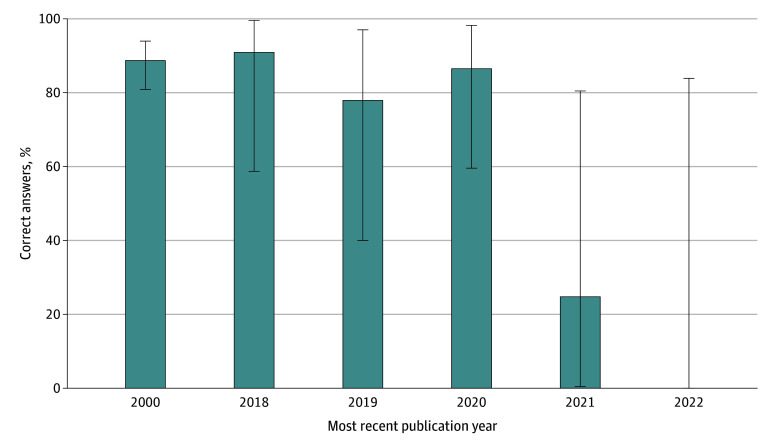
Correct Answers to Medical Oncology Examination-Style Questions by Proprietary Large Language Model 2, by Year of the Most Recent Publication Required to Answer the Question Correctly Error bars indicate 95% CIs.

Incorrect answers were more common when the questions required knowledge of recent publications (Wilcoxon test *P* = .02) (Figure 3): 12 of 106 questions (11.3%; 95% CI, 6.0%-18.9%) requiring only information before 2018 were incorrect, compared with 3 of 20 questions during 2018 and 2019 (15.0%; 95% CI, 3.2%-37.9%), and 7 of 21 questions after 2019 (33.3%; 95% CI, 14.6%-57.0%). Proprietary LLM 2 was trained on data until September 2021, and incorrectly answered both questions (2 of 2 [100%]; 95% CI, 15.8%-100.0%) that required knowledge after that cut-off.

The medical oncologists identified 1 answer from proprietary LLM 2 classified as incorrect that reflects rapidly changing literature and regional practice variations around rectal cancer. For this question on treatment recommendations of a cT3N1 high rectal tumor at 15 cm from the anal verge, proprietary LLM 2 recommended neoadjuvant chemoradiotherapy, while ESMO recommended upfront surgery followed by adjuvant therapy.

The prose accompanying incorrect answers contained the following errors in knowledge recall, reasoning, and reading comprehension (eTable 2 in Supplement 2): 14 of 22 (63.6%) contained errors in knowledge recall alone, while 3 of 22 (13.6%) were due to mistakes in reasoning, and 3 of 22 (13.6%) were due to errors in both recall and reasoning. Only 2 of 22 incorrect answers (9.0%) were associated with erroneous reading comprehension, 1 of which also exhibited errors in knowledge recall. As an example, 1 question asked about a treatment recommendation for a patient diagnosed with metastatic right-sided colon cancer with a *BRAF* V600E variant who achieved a partial response to 5-fluorouracil, oxaliplatin, and bevacizumab but also developed peripheral sensory neuropathy. proprietary LLM 2 recommended switching to targeted therapy instead of maintaining the same chemotherapy regimen without oxaliplatin, which we classified as an error in reasoning.

Among incorrect answers, clinicians considered the likelihood of the error causing patient harm in practice to be medium in 14 of 22 questions (63.6%; 95% CI, 43.0%-85.4%) and high in 4 of 22 questions (18.2%; 95% CI, 5.2%-40.3%). The explanations provided by proprietary LLM 2 contained no or minor errors for 138 of 147 questions (93.9%; 95% CI, 88.7%-97.2%). Incorrect responses were most commonly associated with errors in information retrieval, particularly with recent publications, followed by erroneous reasoning and reading comprehension. If acted upon in clinical practice, 18 of 22 incorrect answers (81.8%; 95% CI, 59.7%-94.8%) would have a medium or high likelihood of moderate to severe harm. Clinicians rated the extent of possible harm as mild in 4 of 22 questions (18.2%; 95% CI, 5.2%-40.3%), moderate in 14 of 22 questions (63.6%; 95% CI, 43.0%-85.4%), and likely to be severe or cause death in 4 of 22 questions (18.2%; 95% CI, 5.2%-40.3%). Whenever there was a high likelihood of harm, the extent was severe, and the same for low likelihood and mild extent. Returning to the previous incorrect answer as an example (Box), we deemed proprietary LLM 2’s recommendation to abandon effective therapy to have a medium likelihood and moderate possible extent for harm, given the recommended course of action could lead to suboptimal treatment of the cancer but does not have immediate life-threatening implications. Four answers were rated as having a high likelihood of harm with potential for severe or life-threatening harm. For example, 1 ESMO question concerned a patient with muscle-invasive bladder cancer who presented with acute kidney injury, whose creatinine clearance only recovered to 38 mL/min with nephrostomy tubes. ChatGPT’s answer recommended neoadjuvant cisplatin-based chemotherapy, which is the standard approach but would be dangerous in a patient with substantially reduced kidney function.

## Discussion

In this study, LLMs performed remarkably well on medical oncology examination-style questions designed for the final stages of training before entering clinical practice. The most recent version, proprietary LLM 2, correctly answered 85.0% of multiple-choice questions and provided accurate written explanations supporting the answers. These results demonstrate that LLMs contain substantial medical oncology knowledge and can provide written responses demonstrating comprehension, retrieval, and reasoning. However, the incorrect answers raise critical safety concerns particularly relevant to medical oncology, where dynamic evidence informs decisions with high-stakes consequences. The explanations accompanying incorrect answers from proprietary LLM 2 showed errors in information retrieval, especially with recent publications. If acted upon in clinical practice, most incorrect answers from proprietary LLM 2 displayed a potential for harm.

Proprietary LLM 2 answered 85.0% of medical oncology questions correctly, the highest proportion reported on medical examination questions to date. Proprietary LLM 2 substantially outperformed its earlier version, proprietary LLM 1. This improvement may explain some of the variation in performance observed across examinations from other medical subspecialties, where proprietary LLM 1 and other LLMs answered 45% to 77% of questions correctly,^6,7,8,9,11,35^ while proprietary LLM 2 answered 57% to 84% correctly.^10,12^ Our results extend beyond a recent study^36^ where proprietary LLM 1 correctly answered 56.1% (583 of 1040) of questions on the ASCO Oncology Self-Assessment Series. We included ESMO examination questions, as well as original questions that could not be in the training set. We also characterized the errors and estimated their potential for harm. Finally, we included proprietary LLM 2 and open-source LLMs. Extrapolating these results, the capabilities of LLMs will likely continue to improve rapidly with larger training datasets and models^37^ and other technical advances.

The performance of LLMs was comparable with medical oncology trainees. According to the annual Examination Report by ESMO, in 2022, the passing threshold was 53.2% correct answers, with the average candidate achieving 60.7% (standard deviation of 11.8%). ASCO does not release summary scores for the ASCO Oncology Self-Assessment Series on ASCO Connection. However, as a comparison, one author participated in the 2019 ASCO In-Training exam as a trainee and answered 35% to 88% of questions correctly across different topics, ranking at the 91st percentile.

The incorrect answers from ChatGPT were most commonly associated with explanations that contained errors in information retrieval, particularly with more recent publications. Errors in retrieving recent information may be intrinsic to the current LLMs because the pretraining procedures weight all text in the training data equally, without specific emphasis on more recent or high-quality information.^27^ We could not refocus the model on the most recent evidence through prompt engineering.^38^ Furthermore, ChatGPT training data only included information up until September 2021. New training procedures and frequent updates may be required for LLMs to provide up-to-date medical oncology information.

Open-source LLMs offer potential advantages over proprietary ones, including lower costs and opportunities for customization. On these examination questions, the best open-source LLM, Mixtral-8x7B-v0.1, was comparable with the proprietary LLM 1 but inferior to proprietary LLM 2. Open-source LLMs can be customized through pretraining, such as with personal health information or fine-tuning for specific tasks, such as mortality prediction.^39^ Surprisingly, BioMistral-7B DARE,^30^ an LLM that pretrained the Mistral foundation model on PubMed Central, underperformed. Together, these results demonstrate a current gap between open-source and proprietary LLMs and highlight the need for research on how to pretrain or fine-tune models for specific health care tasks.

We evaluated LLMs on examination questions as a proxy for their potential when applied to medical oncology more broadly. Proposed use cases include drafting responses to patient inbox messages,^40^ generating clinical reports from patient encounters,^41^ as well as other research and administrative activities.^42^ How our results extend to specific clinical use cases requires further research. Of note, we rated most incorrect answers from proprietary LLM 1 and 2 as having at least a medium likelihood of causing harm, with the possible harm caused as being at least moderate in severity. These results contrast with previous research in general medicine, where clinicians mostly rated responses from LLMs as having a low likelihood of harm.^7^ These findings suggest that the safe use of LLMs in medical oncology may be restricted to low-risk settings or require intensive human oversight. Future research should evaluate techniques to improve safety, such as retrieval augmented generation^43^ and fine-tuning with human feedback.^44,45^ Guidelines should also be developed to promote the safe deployment of LLMs in medical oncology.^46,47^

### Limitations

Our study has several limitations. First, classifications such as the severity and cause of the errors and the likelihood and extent of potential harm are subjective. While our ratings showed high interrater concordance, we acknowledge that others could reasonably disagree with specific ratings. Second, the training data used to create proprietary LLM 1 and 2 is private, so we cannot rule out the possibility that training included the ASCO and ESMO examinations. However, we observed similar results on a set of original questions created for the study, making the performance observed in this study unlikely to reflect memorization during LLM training. Third, our analysis of incorrect answers was based on a small sample size because proprietary LLM 2 made few errors. Fourth, we considered popular and performant LLMs available when the manuscript was drafted, so whether results will generalize to future LLMs is unknown, considering that LLMs are rapidly improving and results from LLMs change over time.^48^

## Conclusions

In this cross-sectional study of the performance of LLMs or examination-style questions in medical oncology, the remarkable performance of proprietary LLM 1 and 2 suggest an urgent opportunity to improve the practice of medical oncology. Potential applications of LLMs span the clinician experience, direct patient care, education, and research.^49^ Our results should encourage oncologists and researchers to develop LLM-based tools within specific workflows and evaluate their impact on measurable outcomes, paying particular attention to safety.^50^

## Notes

Supplement 1.
eMethods.

eReferences.
**eTable 1. **Classification of Errors in Explanations Accompanying Incorrect Multiple-Choice Answers by Large Language Model **eFigure. **Percentage of Correct Multiple-Choice Answers on Medical Oncology Examinations Across Large Language Models

Supplement 2.
eTable 2.

Supplement 3.
Data Sharing Statement
