# Supplementary material for: Performance of Large Language Models on Medical Oncology Examination Questions
Source: JAMA Netw Open. 2024 Jun 18;7(6):e2417641. doi: 10.1001/jamanetworkopen.2024.17641 (PMC11185976; doi:10.1001/jamanetworkopen.2024.17641)
Supplement: Supplement 1. — eMethods. eReferences. eTable 1. Classification of Errors in Explanations Accompanying Incorrect Multiple-Choice Answers by Large Language Model eFigure. Percentage of Correct Multiple-Choice Answers on Medical Oncology Examinations Across Large Language Models [file jamanetwopen-e2417641-s001.pdf]

Supplemental Online Content

Longwell JB, Hirsch I, Binder F, et al. The performance of large language models on medical oncology examinations. *JAMA Netw Open*. 2024;7(6):e2417641.
doi:10.1001/jamanetworkopen.2024.17641

eMethods.
eReferences.
eTable 1. Classification of Errors in Explanations Accompanying Incorrect Multiple-Choice Answers by Large Language Model
eFigure. Percentage of Correct Multiple-Choice Answers on Medical Oncology Examinations Across Large Language Models

This supplemental material has been provided by the authors to give readers additional information about their work.

© 2024 Longwell JB et al. *JAMA Network Open.*

eMethods

Prompting Techniques

ChatGPT was prompted by copying and pasting the questions and potential answers into the web-based user interface. If ChatGPT did not explicitly select one of the multiple-choice options in its response, the phrase "Please select only one of the stated options" was added to the end of the query.

In secondary analyses, we explored ChatGPT's performance after a wrong answer with the prompt: "What would your second-choice answer to the most recent multiple-choice question be?"

We also evaluated whether prompt engineering techniques could improve performance, including chain-of-thought[1], generated knowledge[2], and a novel method of asking the chatbot to use the most up-to-date information. Specifically, we added the phrase before queries: "You are an expert medical oncologist at a top cancer center. Answer the following multiple-choice question. Consider the correctness of each possible answer step by step before selecting the best response. Use the most recent available medical literature and evidence to generate your response."

Open-Source Large Language Models

We evaluated six open-source LLMs with publicly available weights that were ranked highly on Chatbot Arena[3]: Mistral-7B-Instruct-v0.2[4], Mixtral-8x7B-v0.1[5], Llama-2-13b-chat[6], Nous-Hermes-Llama2-70b[7], openchat-3.5-1210[8], and BioMistral-7B DARE[9]. BioMistral-7B DARE is tailored for biomedical domains.

Each model was in the GPT-Generated Unified Format (GGUF) for efficient inference[10,11]. Q4_K_M quantization was employed to reduce resource usage without significantly impacting performance[11–13]. Model inferences were conducted using llama.cpp[14]. Maximum message length, context size, and layers offloaded to the GPU were individually adjusted for each model to optimize performance. All other parameters remained at their default settings. The experiments used a uniform hardware setup consisting of a single GPU and CPU to ensure computational consistency. Most models were instruction-tuned, except for BioMistral-7B. Prompts for each model were formatted according to the specific instruction format specified by each model. For example, the prompt for Mistral-7B-Instruct-v0.2 and Mixtral-8x7B-v0.1 are encapsulated within [INST] and [/INST]. Outputs from the models were standardized to a lowercase letter using neural-chat-7b-v3-1[15] with llama.cpp, employing few-shot learning to ensure uniformity in response. Jupyter notebook was developed to identify and resolve instances of multiple responses within a single output, instructing the models to generate a singular, clear response. A Python script was used to compare the standardized answers against an established answer key, enabling the calculation of each model's accuracy, which was manually verified.

eReferences

1. Wei J, Wang X, Schuurmans D, et al. Chain-of-thought prompting elicits reasoning in large

© 2024 Longwell JB et al. *JAMA Network Open.*

language models. Koyejo S, Mohamed S, Agarwal A, Belgrave D, Cho K, Oh A, eds. *arXiv [csCL]*. Published online January 27, 2022:24824-24837. https://proceedings.neurips.cc/paper_files/paper/2022/file/9d5609613524ecf4f15af0f7b31abca4-Paper-Conference.pdf

2. Liu J, Liu A, Lu X, et al. Generated Knowledge Prompting for Commonsense Reasoning. *arXiv [csCL]*. Published online October 15, 2021. http://arxiv.org/abs/2110.08387

3. Chiang WL, Zheng L, Sheng Y, et al. Chatbot Arena: An Open Platform for Evaluating LLMs by Human Preference. *arXiv [csAI]*. Published online March 7, 2024. http://arxiv.org/abs/2403.04132

4. Jiang AQ, Sablayrolles A, Mensch A, et al. Mistral 7B. *arXiv [csCL]*. Published online October 10, 2023. doi:10.48550/ARXIV.2310.06825

5. Jiang AQ, Sablayrolles A, Roux A, et al. Mixtral of Experts. *arXiv [csLG]*. Published online January 8, 2024. http://arxiv.org/abs/2401.04088

6. Touvron H, Martin L, Stone K, et al. Llama 2: Open Foundation and Fine-Tuned Chat Models. *arXiv [csCL]*. Published online July 18, 2023. http://arxiv.org/abs/2307.09288

7. NousResearch/Nous-Hermes-Llama2-70b · Hugging Face. Accessed April 14, 2024. https://huggingface.co/NousResearch/Nous-Hermes-Llama2-70b

8. Wang G, Cheng S, Zhan X, Li X, Song S, Liu Y. OpenChat: Advancing Open-source Language Models with Mixed-Quality Data. *arXiv [csCL]*. Published online September 20, 2023. http://arxiv.org/abs/2309.11235

9. Labrak Y, Bazoge A, Morin E, Gourraud PA, Rouvier M, Dufour R. BioMistral: A Collection of Open-Source Pretrained Large Language Models for Medical Domains. *arXiv [csCL]*. Published online February 15, 2024. http://arxiv.org/abs/2402.10373

10. GGUF. Accessed April 15, 2024. https://huggingface.co/docs/hub/en/gguf

11. Gerganov G. *Docs/gguf.md at Master · Ggerganov/ggml*. Github Accessed April 15, 2024. https://github.com/ggerganov/ggml/blob/master/docs/gguf.md

12. Gerganov G. *Llama.cpp*. Github Accessed April 15, 2024. https://github.com/ggerganov/llama.cpp/pull/1684

13. Tuggener L, Sager P, Taoudi-Benchekroun Y, Grewe BF, Stadelmann T. So you want your private LLM at home?: a survey and benchmark of methods for efficient GPTs. In: *11th IEEE Swiss Conference on Data Science (SDS), Zurich, Switzerland, 30-31 May 2024*. ZHAW Zürcher Hochschule für Angewandte Wissenschaften; 2024. https://digitalcollection.zhaw.ch/handle/11475/30279

14. Gerganov G. *Llama.cpp: LLM Inference in C/C*++. Github Accessed April 15, 2024. https://github.com/ggerganov/llama.cpp

15. Intel/neural-chat-7b-v3-1 · Hugging Face. Accessed April 14, 2024. https://huggingface.co/Intel/neural-chat-7b-v3-1

© 2024 Longwell JB et al. *JAMA Network Open.*

eTable 1. Classification of Errors in Explanations Accompanying Incorrect Multiple-Choice Answers by Large Language Model

	Before 2018	2018	2019	2020	2021	2022
Total Errors	12	1	2	2	3	2
Extent of error						
Major error	6	1	1	0	0	1
Minor error	6	0	1	2	3	1
Type of error						
Reasoning	4	0	1	0	0	1
Retrieval	9	1	1	2	3	2
Comprehension	2	0	0	0	0	0

© 2024 Longwell JB et al. *JAMA Network Open.*

eFigure. Percentage of Correct Multiple-Choice Answers on Medical Oncology Examinations Across Large Language Models

Random reflects expected performance by random guessing. All models except ChatGPT-3.5 and Chat-GPT-4 are open-source.

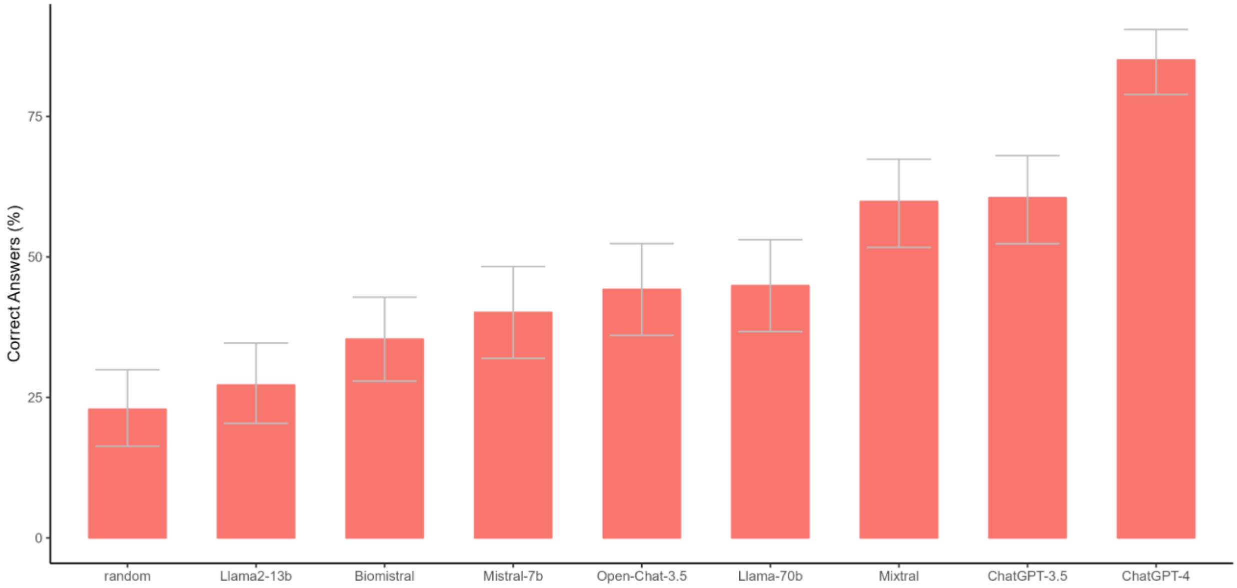

© 2024 Longwell JB et al. *JAMA Network Open.*
