# Supplementary material for: Performance of Large Language Models on Medical Oncology Examination Questions
Source: JAMA Netw Open. 2024 Jun 18;7(6):e2417641. doi: 10.1001/jamanetworkopen.2024.17641 (PMC11185976; doi:10.1001/jamanetworkopen.2024.17641)
Supplement: Supplement 3. — Data Sharing Statement [file jamanetwopen-e2417641-s003.pdf]

Data Sharing Statement

Longwell. The Performance of Large Language Models on Medical Oncology Examinations. *JAMA Netw Open*. Published June 18, 2024. doi:10.1001/jamanetworkopen.2024.17641

Data
Data available: Yes
Data types: Data (not involving human participants)
How to access data: Provided as supplementary material
When available: With publication

Supporting Documents
Document types: Other (please specify)
Additional Information: Supplement with all of the questions reviewed
How to access documents: robert.grant@uhn.ca
When available: With publication

Additional Information
Who can access the data: Researchers whose proposed use of the data has been approved
Types of analyses: Any purpose
Mechanisms of data availability: With investigator support
